# Network motifs and their origins

**DOI:** 10.1371/journal.pcbi.1006749

**Published:** 2019-04-11

**Authors:** Lewi Stone, Daniel Simberloff, Yael Artzy-Randrup

**Affiliations:** 1 Biomathematics Unit, School of Zoology, Faculty of Life Sciences, Tel Aviv University, Israel; 2 Mathematical Sciences, School of Science, RMIT University, Melbourne, Australia; 3 Department of Ecology and Evolutionary Biology, University of Tennessee, Knoxville, Tennessee, United States of America; 4 Department of Theoretical and Computational Ecology, IBED, University of Amsterdam, Amsterdam, the Netherlands; 5 Institute of Advanced Study, University of Amsterdam, Amsterdam, the Netherlands; National Cancer Institute, United States of America and Tel Aviv University, Israel, UNITED STATES

## Abstract

Modern network science is a new and exciting research field that has transformed the study of complex systems over the last 2 decades. Of particular interest is the identification of small “network motifs” that might be embedded in a larger network and that indicate the presence of evolutionary design principles or have an overly influential role on system-wide dynamics. Motifs are patterns of interconnections, or subgraphs, that appear in an observed network significantly more often than in compatible randomized networks. The concept of network motifs was introduced into Systems Biology by Milo, Alon and colleagues in 2002, quickly revolutionized the field, and it has had a huge impact in wider scientific domains ever since. Here, we argue that the same concept and tools for the detection of motifs were well known in the ecological literature decades into the last century, a fact that is generally not recognized. We review the early history of network motifs, their evolution in the mathematics literature, and their recent rediscoveries.

Complex networks now feature prominently in many aspects of modern science and society [1–18]. Within this still rapidly growing discipline, there is strong recognition that large-scale dynamical properties of a network are governed by its much smaller constituent “network motifs,” and so the chief focus is often on motifs. In more technical terms, motifs may be defined as “patterns of interconnections (or subgraphs) occurring in complex networks at numbers significantly higher than those in randomized networks” [1]. Their presence indicates the operation of underlying nonrandom structural or evolutionary design principles that might have been involved in building the network. In a key paper by Milo and colleagues (2002) [1] entitled “Network motifs: Simple building blocks of complex networks,” the authors propose a powerful technique for identifying nonrandom motifs that might otherwise remain hidden. The ability to detect network motifs has had far-reaching scientific impact, and the article of Milo, Alon and colleagues [1] and its three associated papers [2–4] are considered transformative in the field, as can be gauged from the approximately 860 citations they receive annually. However, it is not well known that the very same method used by Milo, Alon and colleagues [1] has a long history in the ecological and social sciences, as discussed here.

In practice, motifs arise in different contexts with diverse forms, as seen in Fig 1 showing a checkerboard motif used in the study of ecological networks [5] (and discussed in more detail below), a clustering triangular motif used in sociological and epidemiological contexts [6–9], and a feed-forward loop motif commonly used in systems biology [1,3,10]. These are only a few examples. Algorithms that detect overabundant motifs have had many applications in systems biology in which they have been used, for example, in the search for regulatory algorithms [4] of the cell and for applications concerning cancer diagnosis [11]. The same techniques are also being applied to study neuronal networks [12], brain function [13], social networks [14], financial [15] and trade networks [16], and internet and mobile wireless communication [17]. This has led to a whole range (quite likely hundreds) of software toolboxes or one-off programs for detecting network motifs [18]. This paper outlines briefly the origins and history of network motifs and the main algorithm for identifying motifs, that is, before its recent “rediscovery” [19] (Merton 1961) at the turn of the millennia by Milo and colleagues (2002) [1] and Shen-Orr and colleagues (2002) [3].

**Fig 1:**
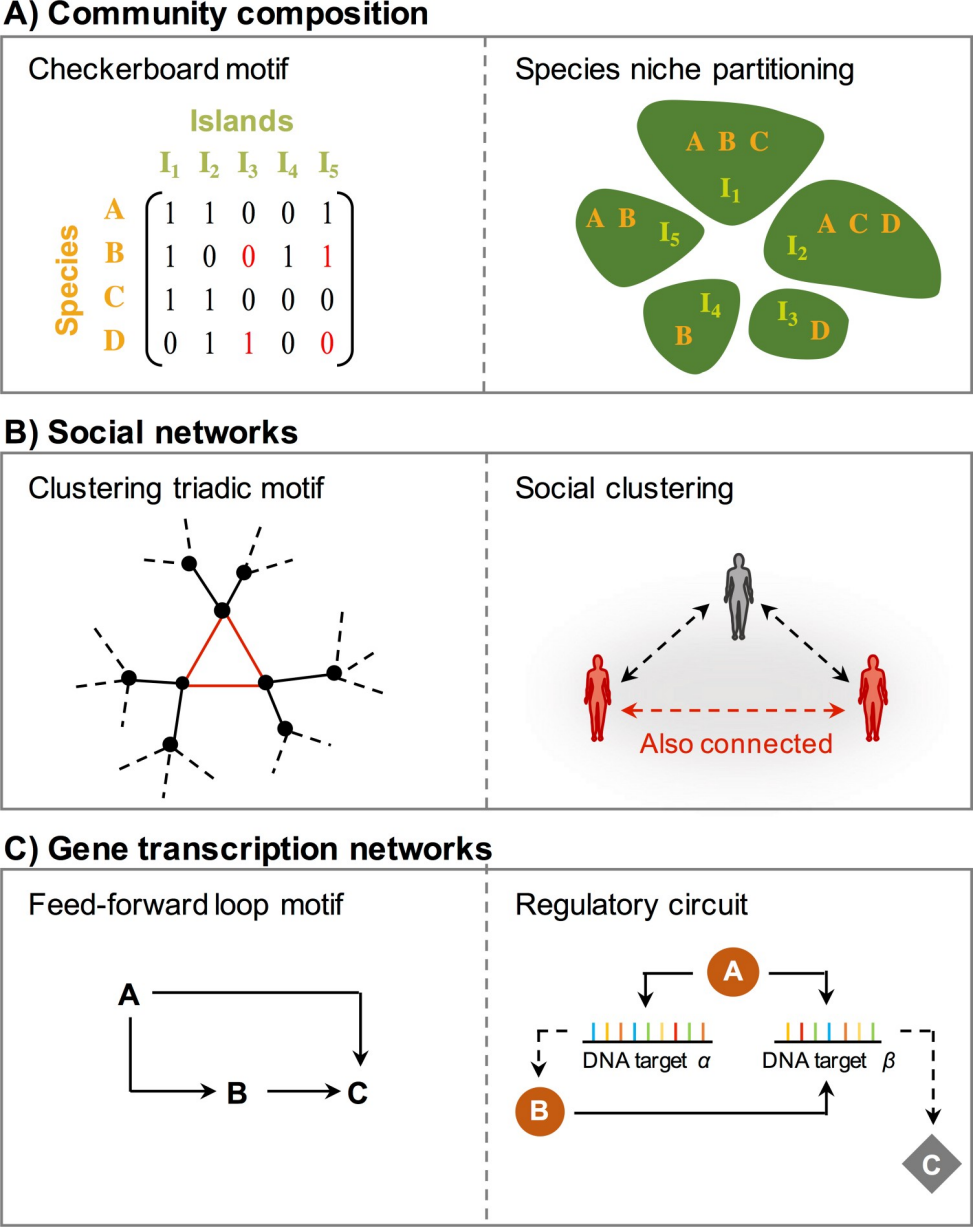
Network motif examples. Motifs in different contexts (right column) and example systems (left column). (A) Checkerboard motif. For example, 4 species (A–D) occupy 5 islands (*I*_1_–*I*_5_). The checkerboard motif highlighted in red represents 2 species that do not co-occur on the same island (here, B appears on *I*_*5*_ but D does not, and conversely, D appears on *I*_*3*_ but B does not), suggestive of competitive interactions. (B) Triadic clustering motif. For example, the motif represents cases in which an individual’s connected friends are also connected with each other, having significance, for example, in social networks and epidemiological contact networks. (C) Feed-forward loop motif. For example, a circuit in gene transcription networks, in which DNA target *β* can be activated only through simultaneous binding of two transcription factors A and B, and in which B depends on A initially binding to DNA targets *α* and *β*, suggesting regulatory control on transcription.

In order to proceed, we define a few basic terms from network theory. Any network or graph may be studied in terms of its binary adjacency matrix *A*. For a network with *n* nodes, and an *n* × *n* binary adjacency matrix *A*, then *A*_*ij*_ = 1 implies that node-*i* is connected to node-*j*, and *A*_*ij*_ = 0 otherwise. The network is undirected if *A*_*ij*_ = *A*_*ji*_, but in general, we deal with directed networks in which this equality usually doesn’t hold. The row and column sums of *A* are given by $r_{i}=\sum_{j=1}^{n}A_{ij}$, and $c_{j}=\sum_{i=1}^{n}A_{ij}$, and represent the in- and out-degrees of all nodes in the network. A key goal is to find a way to generate independent random samples from the full “universe” of all possible binary adjacency matrices that have the same row and column sums *r =* (*r*_*i*_), *c* = (*c*_*j*_), respectively. This universe of matrices is referred to as *U*(*r*,*c*) and constitutes the universe of all possible matrices having the same row and column constraints, thus preserving an important topological feature of the observed adjacency matrix *A*.

In ecological applications, let us suppose for a given adjacency matrix *A* that rows represent species and columns represents islands. Then *A*_*ij*_ = 1 implies that species-*i* inhabits island-*j*. The random matrix ensemble should lock-in characteristics that reflect the ability of some species to colonize islands better than other species as well as the feature that some islands hold more species than others. For this reason, we generate a reference ensemble of random matrices for our null-model in which the row sums (reflecting species colonization abilities) and column sums (reflecting island species numbers) never change [20].

To our knowledge, the first rigorous methods for detecting nonrandom patterns in adjacency matrices or networks, when compared to the universe *U*(*r*,*c*) of all possible matrices, can be traced to the works of Connor and Simberloff (1979) [20], Stone (1988) [21], and Stone and Roberts (1992) [5]. These studies use the so-called switch method to generate an ensemble of random matrices. The method randomly switches or interchanges checkerboard configurations, as shown in Fig 2, and rests on the observation that applying a single such switch leaves the row and column sums of the matrix unchanged. Applying enough switches randomizes the adjacency matrix, but with each switch the row and column sums of the matrix remain preserved. The latter are generally fixed to the values of the observed matrix being tested. Simberloff (1986) [22] and Stone (1988) [21] attempted to show computationally that the switch method randomly samples the universe of all possible matrices from *U*(*r*,*c*) in a manner that is approximately uniform and discussed schemes for drawing samples after every *k* successive interchanges. Zaman and Simberloff (2002) [23] and Artzy-Randrup and Stone (2005) [24] show rigorously, using different methods, that exact uniformity can be achieved with easily implemented weighting schemes. Independent matrix samples so generated from the ensemble *U*(*r*,*c*) provide a reference frame that can be used to estimate motif frequencies that should be expected with a random model. Other novel sampling methods have since been devised [25,26].

**Fig 2:**
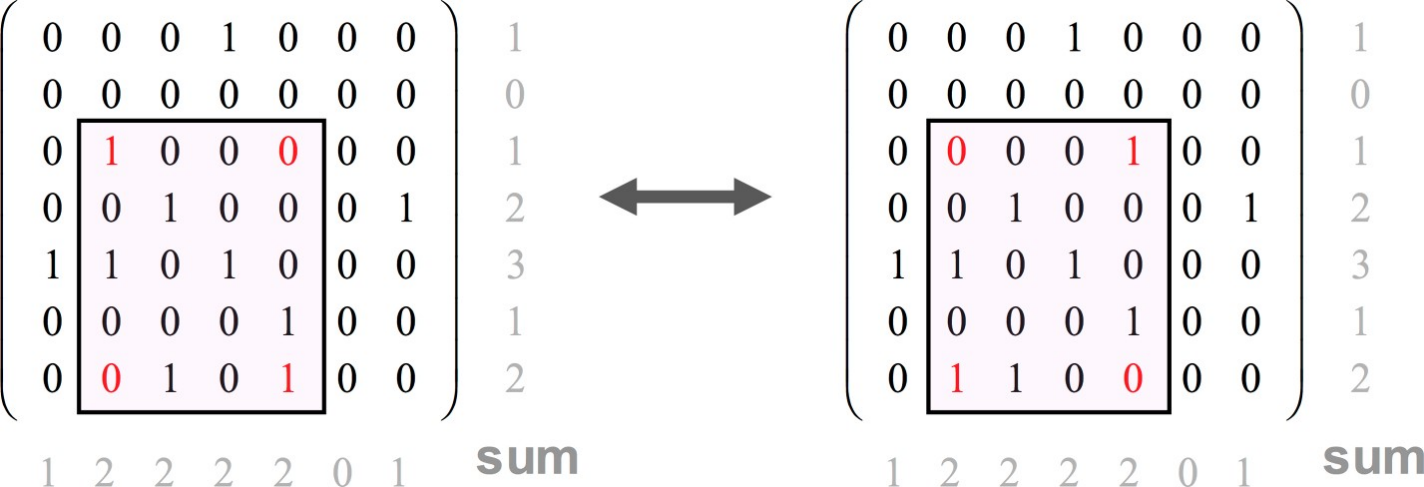
Randomizing matrices with switches. Switches between one checkerboard configuration to another (see 0s and 1s marked in red) leave the row and column sums of the matrix unchanged. One method to generate a set of random samples from the universe of all possible matrices *U*(*r*,*c*) simply requires implementing a large set of switches to randomly chosen checkerboard configurations in the adjacency matrix.

A method specifically for finding an over-represented “network motif” was, to our knowledge, first outlined explicitly in Stone (1988) [21] and Stone and Roberts (1992) [5]. They defined the *C* score as the average number of checkerboard units or motifs between a typical pair of nodes or species. Figs 1A and 2 help explain the concept. Again, the rows of the adjacency matrix *A* represent species and columns represent islands. In Fig 1A, an example checkerboard motif represents a subset of two species (here, B and D) and two islands (*I*_*3*_ and *I*_*5*_). In this case, B does not appear on *I*_*3*_ whereas D does, and although B does appear on *I*_*5*_, *D* does not, suggesting competitive interactions. Fig 2 gives an adjacency matrix in which in the first two rows (species-1 and species-2) have no checkerboard motifs, whereas species-3 and species-4 (third and fourth rows) have two checkerboards. The *C* score of the observed matrix, *C*_*obs*_, is the average number of checkerboard motifs per species pair, when examined for all species pairs. The method then requires finding *μ*, the average of the *C* scores in the ensemble of random matrices, and their standard deviation *σ*. These statistics allow determination of how many standard deviations the observed *C* score is from the mean, namely,

$$z=(C_{obs}-\mu )/\sigma .$$

A large value of *z* (e.g., *z* > 1.96) indicates the checkerboard motif is overrepresented in the network, relative to that expected by chance. This is identical to the method for studying overrepresentation of motifs described by Milo and colleagues (2002) [1] more than 10 years later.

Similar ideas were also used even earlier. In the late 1970s, Holland and Leinhardt (1976) [6,27] attempted to identify small-scale social structure using 3-node motifs (triadic structures of Fig 1). Their research, however, was restricted to analyses of specific classes of random matrices (e.g., having row and column sums that are on average all the same constant), rather than samples having the exact network structure *U*(*r*,*c*) particular to *r* and *c*. The latter approach allows analysis of a much wider and very flexible range of network topologies. Chase (1980, 1982) [28,29] also used triadic network motifs to study hierarchical relationships in animal societies, but these studies were restricted to specific classes of random matrices rather than samples from *U*(*r*,*c*).

The random matrix and network motif methods, once introduced in ecology, metastasized into a huge literature covering foodweb theory, community null models, and assembly rules. This corpus multiplied at a still greater rate when Milo and colleagues (2002) [1] unleashed this method for use in systems biology. These two different fields share the same technique but were discovered independently. We should not be surprised. The phenomenon of multiple discovery is not a rarity, and their occurrences are not simply strange coincidences. Robert Merton (1961) [19] goes so far as to argue that multiple discoveries, rather than unique ones, are the most common pattern in science, sometimes decades apart. Examples include calculus (Newton and Leibniz), evolution (Darwin and Wallace), and the atomic-bomb (Szilard and Rotblat; see also https://en.wikipedia.org/wiki/List_of_multiple_discoveries).

With respect to binary matrices and the search for motifs indicating nonrandom structure, it is not surprising that independent research should have arisen at the beginning of the 21st century. Binary matrices and associated graphs have long been subjects of interest in mathematics, tracing back at least as far as Macmahon (1971) [30] and Sukhatme (1938) [31]. A burst of activity by mathematicians in the late 1950s and early 1960s (e.g., [32–35]; cf. [36]) resulted in many theorems about properties of such matrices, including the size of *U(r*,*c)* and locating a “random” subset of *U(r*,*c)*. Purely mathematical explorations continued well beyond that period (e.g., [36–39]).

“Null models” arose as a hot topic in community ecology and biogeography in the 1970s, primarily in the context of controversies over the importance of interspecific competition and how such competition would be manifested in geographic distribution patterns [20,40]. Because available data were generally in the form of presence or absence of particular species at particular sites, it was inevitable that ecologists with a mathematical cast of mind would eventually come to represent them as species-by-site binary matrices, and the question of manifestations of interspecific competition would then reduce to seeking submatrices representing pairs of mutually exclusive species—motifs, in network terminology. An early ecological effort by Pielou and Pielou (1968) [41] came close to representing such data as a binary matrix but instead turned to analyzing the data as a contingency table. Connor and Simberloff (1979) [20] instead first used a binary matrix representation and analysis. Because the subject of interspecific competition was prominent and controversial during that period, it was inevitable that methods for examining such matrices proliferated in the literature, originally largely independently of the mathematical literature. At approximately the same time, exploration of social networks became a major research focus in sociology, leading to a similar attempt to define and enumerate *U(r*,*c)* and to search for nonrandom patterns in observed networks [42,43]. The rise of network theory in several fields then led almost automatically to research on how to identify patterns and thus to the depiction of network motifs (including checkerboard motifs in ecological networks [44]). Because the fields are quite disparate, some of the relevant literature consists of independently inventing the same wheel. As a result, the rediscovery process occurred over several scientific disciplines, both in parallel and out of sync, and over many years as we have outlined here (and as also independently discussed in Fosdick and colleagues (2018) [45] but from the more recent perspective of stub-labeled configuration models). For these reasons, it is not surprising that the general algorithm for detecting network motifs as invented by Milo and colleagues (2002) [1] is almost identical to that developed by Stone (1988) [21] and Stone and Roberts (1992) [5], which in turn has close similarities to the algorithm suggested by Connor and Simberloff (1979) [20] in their study of ecological networks.
